# Editorial: Exploring child abuse from clinical, legal, and forensic perspectives

**DOI:** 10.3389/fped.2026.1963583

**Published:** 2026-08-18

**Authors:** Donato Morena, Emanuela Turillazzi, Vittorio Fineschi

**Affiliations:** 1 Department of Anatomical, Histological, Forensic and Orthopaedic Sciences, Sapienza University of Rome, Rome, Italy; 2 Department of Surgical Pathology, Medical, Molecular and Critical Area, University of Pisa, Pisa, Italy

**Keywords:** child abuse, child maltreatment, child neglect, abusive head trauma, forensic pathology, forensic medicine, child protection, safeguarding

## Introduction

Child abuse and neglect represent a complex and multidimensional public health and social problem whose recognition and management extend well beyond the boundaries of any single professional discipline. The clinical identification of abuse, the interpretation of potentially non-accidental injuries, the assessment of psychological consequences, the investigation of suspected cases, and the protection of children within legal and social systems require the integration of complementary expertise. This Special Issue brings together contributions addressing these different dimensions, ranging from physician education and public health perspectives to family dynamics, psychological consequences, pediatric abusive head trauma (AHT), and forensic investigation.

Taken together, the contributions highlight a common principle: an effective response to child abuse does not begin and end with diagnosis. Recognition is the necessary first step, but it must be followed by appropriate clinical assessment, multidisciplinary interpretation, standardized forensic investigation, and effective safeguarding and prevention strategies.

An important starting point is the ability of healthcare professionals to recognize possible abuse and respond appropriately. Bayraktar et al. investigated the knowledge, awareness, attitudes, and behaviors of family physicians regarding Child Abuse and Neglect (CAaN), with particular attention to diagnostic competence and mandatory reporting. Their findings indicate that targeted educational interventions can improve physicians' knowledge and diagnostic awareness, supporting the role of structured training in strengthening the clinical recognition of child maltreatment. In their accompanying commentary, Kaewpitoon Rattanapitoon et al. emphasize the importance of integrating child abuse education into continuing medical education and routine primary care practice. Although the persistence of behavioral changes over time was not assessed, these contributions reinforce the concept that training should be considered a continuous process aimed at improving professional confidence, diagnostic vigilance, and knowledge of reporting obligations.

This perspective is broadened by Cebeci, who examines the evolution of child abuse and neglect research through a bibliometric and thematic analysis of the social work literature between 2000 and 2025. The findings support a progressive shift from individualized and family-centered conceptualizations toward a broader public health, child protection, and social welfare framework. At the same time, the geographical and institutional concentration of research highlights the need for stronger international collaboration and more equitable development of child-protection research and policies. Thus, prevention begins not only with the recognition of abuse, but also with the ability of healthcare, social, and child-protection systems to identify vulnerability and establish effective pathways for intervention.

Another important dimension is addressed by Hine et al., who investigate parental alienating behaviors (PABs) among young adults in the United Kingdom and report associations with adverse psychological outcomes, including post-traumatic stress symptomatology, major depressive episodes, and suicidal ideation. This contribution broadens the perspective from physical abuse toward the psychological consequences of harmful family dynamics. In clinical and medico-legal contexts, however, it remains important to distinguish observable parental alienating behaviors from the broader and more controversial construct of parental alienation. High-conflict family situations may involve multiple and overlapping factors, including safeguarding concerns, previous violence, reciprocal conflict, and other forms of family dysfunction. Consequently, assessment should be based on the child's experiences, developmental needs, family context, and available evidence rather than on isolated allegations or diagnostic labels.

The two contributions by Concato et al. provide a particularly relevant example of the clinical and forensic complexity involved in suspected child abuse. Their narrative review of pediatric AHT emphasizes the limitations of relying exclusively on the classical diagnostic triad of subdural hemorrhage, retinal hemorrhage, and encephalopathy, or on an isolated shaking mechanism (Concato et al.). Contemporary assessment requires an integrated interpretation of clinical history, neurological findings, neuroimaging, ophthalmological assessment, and other relevant evidence. Their second contribution proposes a standardized forensic workflow incorporating pre-autopsy assessment and neuroimaging, crime-scene evaluation, comprehensive post-mortem examination, and systematic histopathological and immunohistochemical investigation (Concato et al.). Such standardization may improve diagnostic reproducibility and evidentiary integrity while preserving the essential role of expert interpretation. Previous systematic work on retinal hemorrhages has similarly illustrated the importance of interpreting individual pathological findings within the broader clinical and forensic context (1), further highlighting the value of complementary histopathological and immunohistochemical approaches. In this regard, continued methodological development in forensic histopathology may contribute to greater diagnostic consistency, strengthen the scientific basis of forensic investigations, and improve the integration of pathological findings into the overall evidentiary assessment (2, 3).

When considered together, the contributions of this Special Issue reveal a continuum extending from recognition to multidisciplinary assessment, diagnosis, forensic investigation, legal interpretation, protection, and prevention. Each stage depends on the quality of the preceding one. Failure to recognize suspicious findings may prevent appropriate investigation; inadequate clinical characterization may complicate forensic interpretation; poorly standardized forensic procedures may compromise evidentiary value; and an exclusively legal approach detached from the child's clinical and developmental context may fail to address the child's actual needs.

The central challenge, therefore, is not simply to improve the identification of child abuse, but to develop integrated systems in which clinical medicine, mental health, forensic science, social work, and legal institutions operate as interconnected components of child protection. This is particularly important when evidence is incomplete, clinical manifestations are non-specific, histories are inconsistent, or multiple competing explanations are possible.

Several priorities emerge for future research and practice. International harmonization of diagnostic and forensic approaches could reduce variability across jurisdictions and improve the comparability of evidence. Longitudinal and multidisciplinary research is needed to determine whether educational interventions produce sustained changes in clinical practice and to better characterize the developmental trajectories of children exposed to maltreatment. Finally, greater integration between clinical, forensic, social, and legal systems is essential to ensure that diagnostic accuracy translates into effective safeguarding.

Ultimately, the goal of child-protection systems should extend beyond identifying abuse after it has occurred. It should include the early recognition of vulnerability, the reduction of diagnostic uncertainty, the interruption of abusive dynamics, and the prevention of long-term physical and psychological consequences. In this regard, the contributions collected in this Special Issue illustrate how a truly multidisciplinary approach can strengthen not only the diagnosis and forensic assessment of child abuse, but also the broader objective of protecting children and improving their long-term health and wellbeing.
